# Multi-person feature fusion transfer learning-based convolutional neural network for SSVEP-based collaborative BCI

**DOI:** 10.3389/fnins.2022.971039

**Published:** 2022-07-26

**Authors:** Penghai Li, Jianxian Su, Abdelkader Nasreddine Belkacem, Longlong Cheng, Chao Chen

**Affiliations:** ^1^School of Integrated Circuit Science and Engineering, Tianjin University of Technology, Tianjin, China; ^2^Department of Computer and Network Engineering, College of Information Technology, UAE University, Al Ain, United Arab Emirates; ^3^China Electronics Cloud Brain Technology Co., Ltd., Tianjin, China; ^4^Key Laboratory of Complex System Control Theory and Application, Tianjin University of Technology, Tianjin, China

**Keywords:** steady-state visually evoked potential, collaborative BCI, feature fusion, convolutional neural network, transfer learning

## Abstract

**Objective:**

The conventional single-person brain–computer interface (BCI) systems have some intrinsic deficiencies such as low signal-to-noise ratio, distinct individual differences, and volatile experimental effect. To solve these problems, a centralized steady-state visually evoked potential collaborative BCI system (SSVEP-cBCI), which characterizes multi-person electroencephalography (EEG) feature fusion was constructed in this paper. Furthermore, three different feature fusion methods compatible with this new system were developed and applied to EEG classification, and a comparative analysis of their classification accuracy was performed with transfer learning-based convolutional neural network (TL-CNN) approach.

**Approach:**

An EEG-based SSVEP-cBCI system was set up to merge different individuals’ EEG features stimulated by the instructions for the same task, and three feature fusion methods were adopted, namely parallel connection, serial connection, and multi-person averaging. The fused features were then input into CNN for classification. Additionally, transfer learning (TL) was applied first to a Tsinghua University (THU) benchmark dataset, and then to a collected dataset, so as to meet the CNN training requirement with a much smaller size of collected dataset and increase the classification accuracy. Ten subjects were recruited for data collection, and both datasets were used to gauge the three fusion algorithms’ performance.

**Main results:**

The results predicted by TL-CNN approach in single-person mode and in multi-person mode with the three feature fusion methods were compared. The experimental results show that each multi-person mode is superior to single-person mode. Within the 3 s time window, the classification accuracy of the single-person CNN is only 90.6%, while the same measure of the two-person parallel connection fusion method can reach 96.6%, achieving better classification effect.

**Significance:**

The results show that the three multi-person feature fusion methods and the deep learning classification algorithm based on TL-CNN can effectively improve the SSVEP-cBCI classification performance. The feature fusion method of multi -person parallel feature connection achieves better classification results. Different feature fusion methods can be selected in different application scenarios to further optimize cBCI.

## Introduction

Brain–computer interface (BCI) is a human–computer interaction technology that allows people to directly communicate with a computer or control peripheral device without their surrounding muscles (Vaid et al., 2015). This technology is useful for patients with movement disorders and partial brain injuries, as it helps them realize simple operation and communication (Wolpaw et al., 2000). At present, electroencephalography (EEG)-BCI systems mainly include event-related potentials evoked by endogenous events based on cognitive function (Li et al., 2019), visually evoked potentials (VEP) based on visual stimulation (Mary Judith and Baghavathi Priya, 2021), and event-related area synchronization and event-based active motor imagery in the phenomenon of correlation synchronization (Munzert et al., 2009). Steady-state visually evoked potential (SSVEP) is one of the most popular EEG patterns in the field of BCI. Owing to its advantages such as high information transmission rate (ITR), low requirement on user training, and easy evocation, SSVEP is widely applied to various fields such as medical care, industries, communication, smart home, gaming, robotics, and vehicle control (Zhao et al., 2016; Angrisani et al., 2018; Dehzangi and Farooq, 2018; Farmaki et al., 2019; Nayak et al., 2019; Chai et al., 2020; Shao et al., 2020).

Single-person BCI system’s performance is subject to individual differences between users and their physical or mental conditions, and this weakness becomes more prominent as BCI system develops further (Song et al., 2022). In contrast, multi-person-coordinated BCI can better serve the future socialized human–computer interaction and will most certainly dominate this field both in terms of research and application. Studies have shown that increasing the number of users can substantially improve BCI performance (Valeriani et al., 2016). In human behavior research, teams’ performance is always better than that of individuals. The distinction in performance between teams and individuals is even greater when humans acquire diversified skills, judgments, and experiences under time constraints (Katzenbach and Smith, 2015). As single-person EEG signals have significant individual differences, by collecting multi-person EEG signals and fusing these signals in a reasonable way, signals with more distinctive features can be obtained, and the BCI performance can be improved. EEG signals from multiple subjects can significantly improve ITR in the system compared to single EEG signals (Bianchi et al., 2019). Subjects who need to stare at the stimulation area for a long time are prone to fatigue due to visual stimulation in SSVEP-BCI, which affects the quality of EEG signal acquisition, and this is particularly evident for some subjects (Peng et al., 2019). SSVEP-cBCI can make up for this deficiency by increasing the user dimension and improve the information transmission rate. Acknowledging this viewpoint, this paper explores three feature fusion methods, which include (1) parallel connecting features, (2) serial concatenating features, and (3) feature averaging. These approaches will be explained in detail in section “Methods.” The three feature fusion methods aim to improve the signal-to-noise ratio by merging multi-person EEG information to get refined new features to enhance the BCI performance.

As a branch of machine learning, deep learning has achieved great success in solving problems in computer vision and natural language processing. It is different from traditional machine learning as it does not entail manual feature extraction (LeCun et al., 2015). Using gradient descent learning to optimize convolutional neural network (CNN) parameters successfully solved the problem of handwritten digit classification (LeCun et al., 1998). However, owing to the complexity of EEG signals, the application of deep learning neural networks in EEG signal detection is still in the exploratory stage. Cecotti and Graser (2010) developed a four-layer CNN for P300 detection. At present, the SSVEP EEG signal classification method converts the original EEG signal through FFT and then inputs it into CNN for classification (Cecotti, 2011; Zhang et al., 2019; Ravi et al., 2020). As a superb CNN model designed for EEG, EEGNet exhibits good classification performance, but other models perform better in some moments. In this study, some details of the basic EEGNet were adjusted, and the network structure was modified to adapt to the newly created fusion features. The transfer learning (TL) training strategy using a THU benchmark dataset as the source task training set was adopted to initially train the parameters of the convolutional layer and build the basic feature extractor. Using the data collected by the laboratory as the target task training set and test set, the CNN parameters were further optimized to construct SSVEP-cBCI. In this paper, the classification model is trained with the TL-CNN method, which reduces the required amount of training collected data and improves the classification accuracy. And the feature fusion approach further improves BCI performance in classification accuracy, ITR and stability.

Section “Methods” elaborates on the personnel, equipment, and experimental paradigms associated with the experiments, the three multi-person features fusion methods, the specific structure of the modified CNN in this study, and its difference from EEGNet. Then the following part introduces the specific training method of TL. In section “Results,” the classification accuracy and ITR difference of the three feature fusion methods and those predicted by a single-person CNN are compared. Finally, some significant conclusions are drawn, and the specific usage of the three feature fusion methods in this experiment is analyzed.

## Methods

### Experimental setup

#### The structure of cBCI system

**FIGURE 1:**
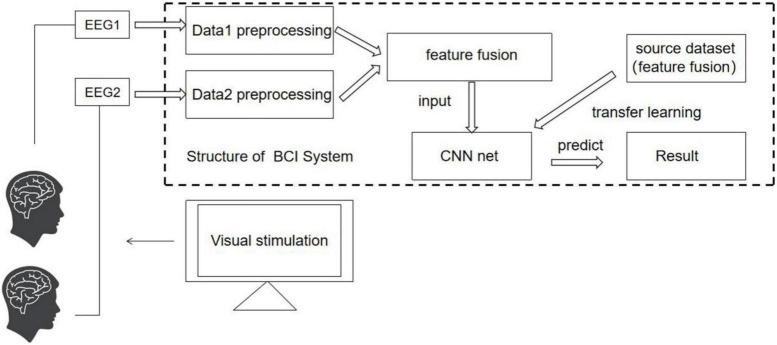
Centralized cBCI structure designed in this study.

The cBCI system mainly has two structural forms: distributed and centralized (Wang and Jung, 2011). In both systems, experiments are simultaneously conducted on more than one subject. In the distributed cBCI, subjects’ EEG information is collected individually for subsequent data preprocessing, feature extraction, and pattern recognition through the corresponding BCI subsystem. The results corresponding to each subject are then transmitted to the integrated classifier, and the final decision is produced through decision-making layer’s voting mechanism, while in the centralized cBCI, as shown in Figure 1, subjects’ EEG information is collected individually for sequential data preprocessing and feature extraction. The EEG data features of all subjects are fused for pattern recognition to make the final decision for the group. The model adopted in this study is a centralized cBCI system, which does not rely on the voting mechanism of the distributed system, and classification is carried out with a CNN based on TL (TL-CNN).

#### Experimental paradigm

In this experiment, the EEG data were collected and transferred from the EEG amplifier to the software Curry8 (Neuroscan). Three electrodes were placed on O1, Oz, and O2 according to the International 10–20 system. Using the double mastoid as reference and ground electrodes, the impedance of all electrodes was reduced to below 5 kΩ. The sampling frequency is 256 Hz, and a band-pass filter between 5 and 40 Hz is used in the data processing to filter out low-frequency noise and 50 Hz power frequency noise.

**FIGURE 2:**
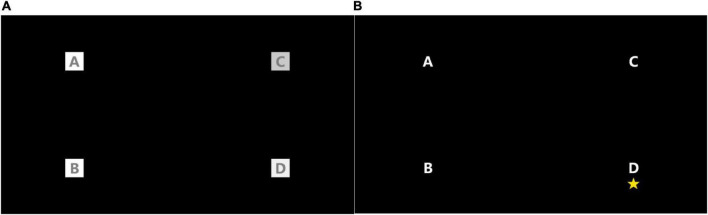
SSVEP stimulation interface and label reminder method. **(A)** Stimulus interface. **(B)** Random tag prompt.

Ten healthy subjects (8 males, 2 females, 21–27 years old) participated in the experiments. All participants had normal or corrected vision. Four of them had participated in SSVEP experiments previously. All participants read and signed the informed consent forms. Subjects sat on a comfortable chair 60 cm in front of a standard 24-inch monitor (60 Hz refresh rate, 1,920 × 1,080 screen resolution). The SSVEP stimulation interface is shown in Figure 2, and the four stimulation squares are all 50 × 50 pixels. The refresh frequency of the display equals integer multiples of the stimulation frequency of the four color blocks, which can ensure stable stimulation frequency and avoid frequency deviation. The stimulation frequencies of the four color blocks are 8.6, 10, 12, and 15 Hz, respectively. It was evidenced that stimulation frequencies of 10 and 12 Hz can stably induce high-amplitude SSVEP signals (Chen et al., 2015), and the stimulation duration was set to be 4 s. To avoid interference caused by simultaneous flickering of the four color blocks, the phases of the four color blocks are set as 1.35π, 0.35π, 0.9π, and 0.35π, respectively. Prolonged staring at the flickering stimulus color blocks made the subjects feel tired and distracted them, resulting in a frequency deviation of the SSVEP signal. To improve the concentration of the subjects and the quality of SSVEP EEG signals, random labels were used to remind the subjects to look at the corresponding stimulus squares.

0.02 s after the five-pointed star appeared, the four color blocks started to flash. After the flashing, a rest time of 2 s was given to the subject to adjust the viewing angle. During the experiment, the subjects were asked to focus on the corresponding color block and blink as few times as possible. Each color block flashed twice in total, and there was a 1-min rest between two consecutive experiments.

### Multi-Person feature extraction and fusion

#### Multi-Person feature extraction

The EEG data filtered and processed by the fourth-order Butterworth filter is converted from the time domain to the frequency domain by FFT transformation (Chen et al., 2015). Low-frequency (8.6, 10, 12, and 15 Hz) stimulation area was used in these experiments. The features of the frequency band from 6 to 32 Hz were selected from the FFT-transformed data to further filter out noise and improve feature quality.

The characteristics of the SSVEP signal are as follows:


(1) $$\left\{ \begin{array}{c} F\,e\,a\,t\,u\,e_{O\,1}=\left|F\,F\,T\,\left(X_{O\,1}\right)\right|\\ F\,e\,a\,t\,u\,e_{O\,Z}=\left|F\,F\,T\,\left(X_{O\,z}\right)\right|\\ F\,e\,a\,t\,u\,e_{O\,2}=\left|F\,F\,T\,\left(X_{O\,2}\right)\right| \end{array}\right.$$

The input of the convolutional neural network is:


(2) $$I\,n\,p\,u\,t=\left|\begin{array}{c} m\,i\,n\,\_\,m\,a\,x\,\left(F\,e\,a\,t\,u\,e_{O\,1}\right)\\ m\,i\,n\,\_\,m\,a\,x\,\left(F\,e\,a\,t\,u\,e_{O\,z}\right)\\ m\,i\,n\,\_\,m\,a\,x\,\left(F\,e\,a\,t\,u\,e_{O\,2}\right) \end{array}\right|$$

The min–max normalization (discrete normalization) is conducted on the data of each channel (Ali et al., 2014) to avoid adverse effects on the classification accuracy owing to huge differences between values, ensure good performance of different data within the same neural network, and improve the robustness of the algorithm.

#### Feature fusion

**FIGURE 3:**
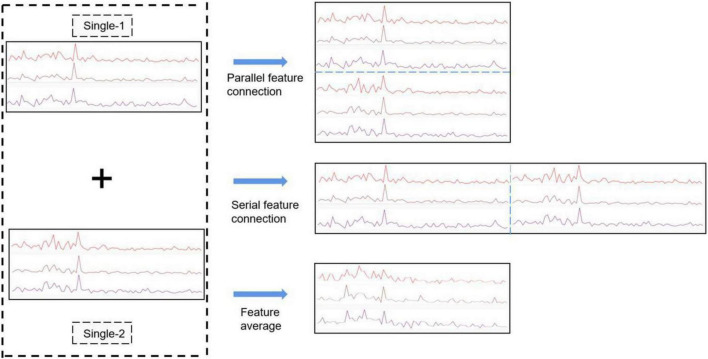
Three fusion methods: (1) feature parallel connection (2) feature serial connection (3) feature averaging. Single-1 and Single-2 represent two single-person features for feature fusion.

This paper proposes three methods to fuse multi-person EEG features. As shown in Figure 3, in parallel connection, three-channel (O1, Oz, O2) data are extracted to obtain the effective feature after data preprocessing and FFT transformation. The features of different subjects are connected in parallel, serial concatenation, or averaging. In parallel feature concatenation, connection is made mainly the spatial domain, which implies more feature lead channels. In serial feature connection, connection is made mainly in the frequency domain, which implies that there is no change in the number of channels, but the domain scope expands greatly, and thus, the effective features are enhanced from the frequency perspective and the BCI performance improves. However, serial concatenation requires more training on epoch and convolution kernel to achieve the similar classification accuracy of parallel connection. It involves more complex algorithm, so it is more difficult to set up an online system by Python. The above two feature fusion methods are suitable for subjects with a known number of participants in the experiment, but when the number of participants in the brain group is unknown, different CNNs meant for various number of subjects should be set up and trained, which entails more input in the experimental preparation. This problem can be solved by adopting the third approach, feature averaging, that is, to get new features by averaging the normalized EEG frequency features of all subjects. The CNN using this approach shares the same structure of single-person CNN, and its classification accuracy is superior to that of a single-person CNN but inferior to that of a two-person CNN.

### Deep learning network construction

#### Improved the network structure of convolutional neural network

Lawhern et al. (2018) designed EEGNet, a compact CNN specially for EEG signals, that is based on CNN and includes two parts: spatial feature extraction and frequency or time domain feature extraction. It can efficiently extract features from frequency-domain EEG information and send them to a neural network-based classifier, eliminating the need to manually extract two-part features. This paper makes some adjustments on the EEGNet, changing the number of convolution kernels, the size of the convolution kernel, and the depth of the convolution layer. In this experiment, the collected data were used to predict the classification accuracy of the test set, and the EEGNet was modified to accommodate the data. In this study, the ordinary convolution layer was discarded, a depthwise convolution layer was added, and the pointwise convolution layer was changed into a small narrow-band convolution. The network consists of six consecutive layers, including four convolutional layers, one fully connected layer, and one softmax output layer (Jang et al., 2016). Network fitting is accelerated through batch normalization (Ba et al., 2016). Linear activation layer adopts ReLu function (Agarap, 2018).

Table 1 summarizes the modified CNN structure. The convolution kernel of the C1 convolution layer has size 3 × 1, and its function is to learn the linear combination and spatial filtering features between different channels. The method of padding and zero-filling is adopted to prevent the loss of information caused by convolution (Dwarampudi and Reddy, 2019). The C2 layer does not use the method of padding and zero-filling and integrates multi-channel data into a single channel by convolution. The C3 convolution layer extracts features along the input frequency spectrum by convolution and acts as a band-pass filter. The C4 convolution layer also integrates frequency features without padding. Among them, the convolution operations of the C2 and C4 layers have achieved the down-sampling effect. After Flatten layer, a fully connected layer is followed by softmax for classification.

**TABLE 1:** Single-person convolutional neural network structure.

Layer number	Layer	Filter	Kernel size	Feature size	Activation
1	Input data	–	–	(3.78)	–
2	Conv2D	16	(3.1)	(3.78)	ReLU
3	Conv2D	32	(3.1)	(1.78)	ReLU
4	Conv2D	32	(1.3)	(1.78)	ReLU
5	Conv2D	64	(1.3)	(1.76)	ReLU
6	Flatten	–	–	–	–
7	Dense	8	–	–	–
8	Dropout	–	Rate = 0.5	–	–
9	Dense	4	–	–	Softmax

#### Network structure of feature fusion convolutional neural network

As can be seen in Table 2, compared with the single-person CNN, the difference between the two-person parallel feature concatenation CNN structure is that it increases the number of key channels, from three-channel to six-channel EEG data, which greatly increases the number of features.

**TABLE 2:** Two-person parallel feature connection CNN structure.

Layer number	Layer	Filter	Kernel size	Feature size	Activation
1	Input data	–	–	(6.78)	–
2	Conv2D	16	(6.1)	(6.78)	ReLU
3	Conv2D	32	(3.1)	(4.78)	ReLU
4	Conv2D	64	(3.1)	(2.78)	ReLU
5	Conv2D	64	(2.1)	(1.78)	ReLU
6	Conv2D	128	(1.3)	(1.78)	ReLU
7	Conv2D	256	(1.3)	(1.76)	ReLU
8	Flatten	–	–	–	–
9	Dense	8	–	–	–
10	Dropout	–	Rate = 0.5	–	–
11	Dense	4	–	–	Softmax

Therefore, the two-person parallel feature connection CNN structure was added to unpadded convolution layers C2 and C3 in accordance with the single-person CNN structure while keeping the fully connected layer and the last two layers unchanged. The network classification results show that the classification accuracy falls by about 1% as each of the two convolutional layers is reduced. Multiple convolution operations can effectively extract complex multi-channel features and integrate them into a single spatial feature.

As can be seen in Table 3. The CNN structure used by the two-person serial feature connection method is similar to the single-person CNN structure. With the dual serial feature connection, the number of features input to the CNN is increased. This builds more feature extractors by increasing the number of convolution kernels to get better results. If the number of convolution kernels of the two-person CNN connected by serial features is the same as that of the single-person CNN, the classification accuracy will drop by about 2%.

**TABLE 3:** Two-person serial feature connection CNN structure.

Layer number	Layer	Filter	Kernel size	Feature size	Activation
1	Input data	–	–	(3.234)	–
2	Conv2D	48	(3.1)	(3.234)	ReLU
3	Conv2D	96	(3.1)	(1.234)	ReLU
4	Conv2D	96	(1.3)	(1.234)	ReLU
5	Conv2D	192	(1.3)	(1.232)	ReLU
6	Flatten	–	–	–	–
7	Dense	8	–	–	–
8	Dropout	–	Rate = 0.5	–	–
9	Dense	4	–	–	Softmax

#### Transfer learning-based feature fusion strategy with different datasets

Compared with traditional machine learning algorithms, deep learning methods heavily rely on high-quality data. Obtaining sufficient high-quality datasets to train high-quality convolution kernel parameters is a critical problem to be solved in CNN setup. Transfer learning (Pan and Yang, 2009) gives an effective solution to this problem. The SSVEP EEGs collected in the THU benchmark dataset (Chen et al., 2015) exhibit good features and low error rates of subjects’ operation, and thus, this dataset was used as the source dataset for initial parameters training on the model. In general, parameters in CNN are randomly initialized by training collected data directly. Compared with transfer learning, it requires a larger amount of data and training time to fit and get a satisfactory feature extractor. While using transfer learning methods, initial parameters can be constructed in a pre-training manner, and these parameters are usually derived from prior knowledge and hence can well perform the corresponding task. As a consequence, only a small amount of actual experimental data serve as the training set, and the model parameters are re-learned through fine-tuning for the model to adapt to the actual experimental data. This method can improve the classification accuracy of the model and effectively reduce the required size of experimental data collected in our laboratory to train the CNN.

**FIGURE 4:**
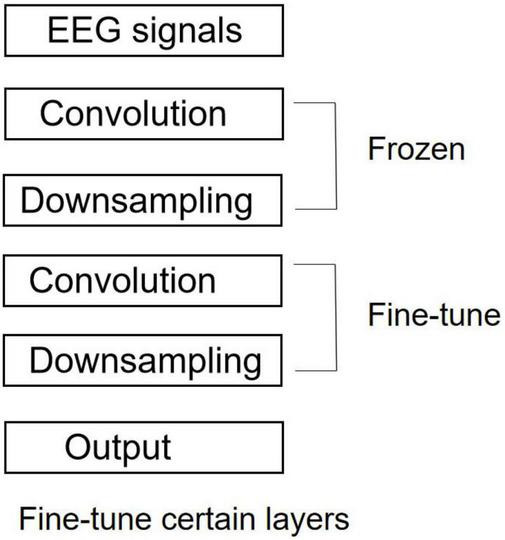
Training strategy.

The comparison among various fine-tune methods suggests significant differences in their stability but insignificant difference in their classification accuracy. Figure 4 shows that only the parameters of the deep convolutional layer and the fully connected layer are trained, while the parameters of the shallow CNN are frozen and not involved in the training. Since the feature distribution of the source task data (THU benchmark dataset) does not coincide with that in this experiment, fine-tuning on the parameters of the deep convolutional layer with a small learning rate can improve the feature extraction performance of the convolutional layer.

In the first step, training was conducted with the THU benchmark dataset as the training set, and a total of 720 samples were used, with 180 samples per stimulus. The network weights are learned in accordance with the Adam learning algorithm, which optimizes the network weights through back-propagation, and the cross-entropy function is adopted as the loss function (Zhang and Sabuncu, 2018).


(3) $$L\,o\,s\,s=-\sum_{j=1}^{T}y_{j}\,l\,o\,g\,P_{j}$$

The data of 24 people in the THU benchmark dataset is used as the pre-training dataset, and different combinations of multiple people are randomly used for feature fusion. After repeated experiments to verify, the different combinations of multiple people used for feature fusion during the pre-training of the initialized feature extractor do not have an impact on the final classifier. An initial pretraining learning rate of 0.001 was adopted. The epoch was set to 80 and the mini-batch size of stochastic gradient descent is set to 16. Next, the pre-training model with initial weights was established for the experimental paradigm followed in this study. Based on the pre-training model, the epoch was then reset as 40 for training with the collected experimental data. A very large epoch makes a personal private network and reduces the generalization ability (Pan and Yang, 2009).

## Results

The 10 subjects were labeled as S1 to S10, and two adjacent subjects made up one group (e.g., S1 and S2 made up group C1, and S3 and S4 made up C2). Table 4 shows only one grouping case to show the fusion of features at different levels of feature quality, group members were interchangeable and tried different combinations. As the parallel feature connection method gives the best classification result with fused features and CNN, Table 4 only lists the classification of different subjects and subject groups in different time windows under parallel feature connection. The three-channel EEG data of the two people in each group were connected in parallel to obtain six-channel EEG data, and the six-channel CNN model was trained using the TL strategy. Table 4 shows that, based on the CNN classifier, the classification accuracy of both single- and two-person feature fusion declines as the time window decreases. Personal characteristics become more marked when the stimulation time is longer. This table compares the classification accuracy results of single-person CNN and five representative results of two grouping types with two people in a group: (1) Feature fusion of subject data with significant and insignificant features. That is, one of them yielded a high classification accuracy, but the other yielded a low classification accuracy. The final result is lower than the best single-person classification accuracy with significant features. (2) Feature fusion of subjects’ data with only significant features. As the data features of the two people were both significant, the classification accuracy of the neural network was markedly improved.

**TABLE 4:** Classification accuracy comparison of single- and two-person models under different time windows.

	TL-CNN (%)							
	3 s	2.8 s	2.6 s	2.4 s	2.2 s	2.0 s	1.8 s	1.6 s
S1	91.6	87.5	81.3	77.1	79.1	75	70.8	60.4
S2	89.5	85.4	87.5	83.2	77.1	66.7	66.7	66.6
S3	97.9	91.6	91.6	89.6	91.7	83.3	77.1	70.8
S4	79.1	68.6	77	70.8	66.7	68.6	70.8	64.5
S5	100	100	97.9	95.8	95.8	93.7	87.5	75
S6	93.7	87.5	83.3	83.3	83.4	79.2	77.1	72.9
S7	79.1	77	77.1	68.8	60.4	60.4	58.3	54.2
S8	100	100	100	97.9	97.9	93.4	87.5	85.4
S9	75	64.5	68	70.9	60.4	60.4	60.4	50
S10	100	97.9	97.9	93.7	91.7	91.7	72.9	72.9
Saverage	90.6	86.4	86.2	82.4	80.4	77.3	72.9	67.3
C1	95.8	93.3	87.5	79.2	91.6	77	75.1	75
C2	89.5	93.3	95.5	85.4	81.2	79.2	81.3	75
C3	100	95.8	97.9	95.8	91.7	85.4	91.2	91.2
C4	100	100	95.8	93.7	87.5	85.4	85.4	75
C5	97.9	97.9	93.7	93.7	83.3	87.5	81.2	70.8
Coverage	96.6	96.1	94.1	89.6	87.1	83.1	82.9	77.4

Taking the 3-s time window as an example, the 10-person average classification accuracy of the single-person system CNN without TL is only 43.5%, but with TL, it can reach as high as 90.6%. The five-person average classification accuracy of the two-person CNN without TL is only 55.0%, while with TL, it reaches 96.6%.

**FIGURE 5:**
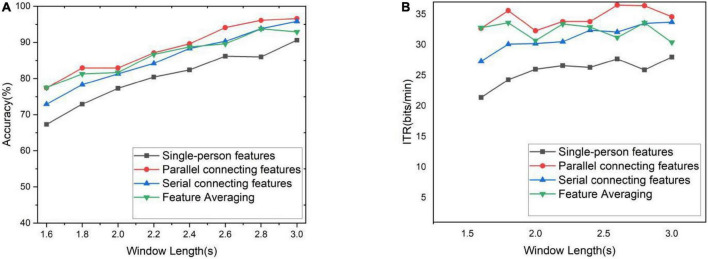
Accuracy and ITR under different time windows. **(A)** Classification accuracy for different time windows. **(B)** ITR for different time windows.

The 10-person data containing S1–S10 were used as data sets for subsequent experiments and called the collected data set. The results of each model training and prediction are different. The collected data are randomly shuffled, and then feature fusion is performed to calculate the average classification accuracy and ITR through the 10-fold cross-validation method, as shown in Figure 5, respectively. It can be clearly seen from Figure 5 that the classification accuracy results of the two feature fusion methods and feature averaging method based on CNN invariably exceed that of single-person CNN in different time windows. Three multi-person fusion methods based on CNN ITR significantly outperformed single-person CNN (*p* < 0.0001). Parallel feature connection ITR also significantly outperformed the other two feature fusion methods (*p* < 0.05). Among these three methods, the parallel feature connection method always ranks first, with the highest classification accuracy and ITR. The serial feature concatenation method and the feature averaging method exhibit similar overall performance, but the feature averaging method is more flexible and requires less computation. It can thus be concluded that feature averaging is better than serial feature concatenation.

The optimal scheme to set up cBCI is to train the corresponding parallel feature connection model with the TL method in advance when the number of subjects is known or to train the single-person model with the TL method and then apply the feature averaging method to this trained model when the number of subjects is unknown.

**FIGURE 6:**
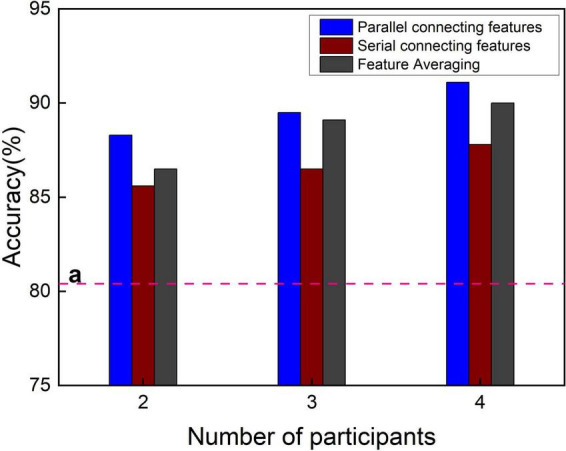
Total classification accuracy of three fusion methods under different number of participants. The line “a” is the total classification accuracy of the single features.

The classification accuracy of different time windows was averaged to obtain the total classification accuracy as shown in Figure 6. The total classification accuracy of the single-person CNN is 80.4% as baseline, which is far lower than the total classification accuracy of the multi-person CNN-based three feature fusion methods. As can be seen from Figure 6, when the number of participants in the experiment increased, the total classification accuracy of the three feature fusion methods slightly improved. The fusion method of parallel feature connection invariably attained the highest total classification accuracy; the feature averaging method was always noted to be the second best, and the serial feature connection was found to be the worst. Why is the parallel method so good? Upon increasing the number of participants, owing to the increase in the number of features, the CNN used by the three feature fusion methods needs to be slightly modified, mainly by increasing the number of convolution kernels. However, as the parallel feature connection needs to continuously integrate the information of multiple-lead channels through convolution, more convolutional layers are added. It has been proven that the convolution method can integrate the features of multiple individuals and multiple leads in a nonlinear way, which is better than the method of feature averaging. Therefore, if the computing power of the computer allows, it is an excellent cBCI construction method to use the method of parallel feature connection to fuse the EEG features of multiple people and send them into the TL-CNN model.

## Discussion

Visually evoked potentials have been extensively studied by researchers (Mary Judith and Baghavathi Priya, 2021). When subjects gaze at flickering visual stimuli with flicker frequencies exceeding 4 Hz, their cerebral cortical activity would be modulated, resulting in a periodic rhythm similar to that of the stimulus (Bondre and Kapgate, 2014). CNN-based EEGNet, which was specially designed for EEG, has been widely applied to classification tasks in various EEG paradigms: e.g., P300 VEP, falsely correlated negatively matched waves, motor-related cortical potentials, and sensorimotor rhythms. In this study, the original EEGNet was modified, and the accuracy of the classification mode was improved by increasing the number of convolutional layers and the number size of convolutional kernel of each convolutional layer. In addition, three different multi-person EEG feature fusion methods are proposed herein to integrate multi-person EEG information to improve BCI performance. Each of the three fusion methods was found to have merits and shortcomings. In summary, in the case of known multi-person BCI collaborations with a fixed number of people, the parallel feature connection method is the best choice because it involves smaller models and fewer training parameters than the serial connection method; also, compared with feature averaging method, it produces higher classification accuracy. When the number of subjects is unknown, the feature averaging method should be chosen, and feature averaging using multiple subjects can be directly applied to a trained single-person CNN. Serial feature concatenation method is not suitable for the construction of online BCI system.

In this study, a small number of leads (e.g., O1, OZ, O2) were collected for setting up a multiple-person BCI system, which can facilitate the experimental preparation, reduce the subjects’ fatigue, and improve the system’s execution efficiency. Different from the voting and averaging methods of the existing distributed multi-person BCI systems, a complete single EEG data is constructed through multi-person feature fusion, and a transfer learning-based CNN is used to achieve classification in this new system. Compared with traditional CNN methods, the number of samples to collect is markedly reduced, and the accuracy is slightly improved. Moreover, a neural network with multiple narrow-band convolution kernels is constructed, and a multi-channel and multi-person feature fusion method is set up to extract the corresponding nonlinear features for fusion so as to improve the recognition accuracy of SSVEP-cBCI, further enhancing the classification accuracy and signal-to-noise ratio. The experimental results of the 10 subjects show that the CNN classification fusing two persons’ features produces a higher SSVEP-cBCI recognition accuracy, and the TL-CNN-based two-person BCI effectively raises the classification accuracy and the robustness of BCI. The impact of individual differences in single-person BCI systems on system performance stability has been resolved. With the increase in the number of participants in the experiment, the total classification accuracy of the three feature fusion methods has been slightly improved, and the parallel feature connection method invariably exhibits the best performance.

The deep learning SSVEP-cBCI algorithm based on multi-person feature fusion established in this paper has been verified through offline system experiments and can be extended to real-time online systems in the future to complete real-time control of external equipment. Since the SSVEP-cBCI experimental paradigm requires multiple subjects to simultaneously fixate on the same flickering stimulus interface, a method of replicating multiple monitors was employed, and the collected multi-person EEG data were used for subsequent data processing and identification by a microcomputer. In the case of a fixed number of multi-person BCI collaborations and the computing power is allowed, it is necessary to prepare multiple corresponding number of different participants CNN classification models, perform corresponding feature fusion (parallel feature connection) and classification model training, and use the trained models to complete real-time online experiments. This feature fusion method can maximize the performance of cBCI. If the number of multi-person BCI collaborations is not fixed, or the computing power is not allowed, or there is not enough corresponding CNN classification model established, then the multi-person features can be integrated by the method of feature averaging, and the single-person CNN model can be used to complete real-time online experiments. Parallel feature connection are suitable for high-precision tasks, such as controlling unmanned vehicles, which requires precise control of the vehicle’s travel to avoid collision. The feature averaging method fits into rehabilitation centers. Different numbers of patients can send requests at the same time, and as the number increases, medical staff can make corresponding responses more accurately. Serial feature connection can be used as an alternative method to increase the robustness of cBCI.

## Data Availability

The raw data supporting the conclusions of this article will be made available by the authors, without undue reservation.

## References

[B1] Agarap A. F. (2018). Deep learning using rectified linear units (relu). *arXiv* [Preprint] arXiv:1803.08375 10.48550/arXiv.1803.08375

[B2] Ali P. J. M. Faraj R. H. Koya E. Ali P. J. M. Faraj R. H. (2014). Data normalization and standardization: a tec‘hnical report. *Mach. Learn. Tech. Rep.* 1 1–6. 10.13140/RG.2.2.28948.04489

[B3] Angrisani L. Arpaia P. Moccaldi N. Esposito A. (2018). “Wearable augmented reality and brain computer interface to improve human-robot interactions in smart industry: a feasibility study for ssvep signals,” in *Proceedings 2018 IEEE 4th International Forum on Research and Technology for Society and Industry (RTSI)*, (Piscataway, NJ: IEEE), 1–5. 10.1109/RTSI.2018.8548517

[B4] Ba J. L. Kiros J. R. Hinton G. E. (2016). Layer normalization. *arXiv* [Preprint] arXiv:1607.06450 10.48550/arXiv.1607.06450

[B5] Bianchi L. Gambardella F. Liti C. Piccialli V. (2019). “Group study via collaborative BCI,” in *Proceedings of the 2019 IEEE International Conference on Systems, Man and Cybernetics (SMC)*, (Piscataway, NJ: IEEE), 272–276. 10.1109/SMC.2019.8914482

[B6] Bondre C. Kapgate D. (2014). Brain computer interfaces using SSVEP: an overview. *Int. J. Eng. Technol.* 1 9–17.

[B7] Cecotti H. (2011). A time–frequency convolutional neural network for the offline classification of steady-state visual evoked potential responses. *Pattern Recog. Lett.* 32 1145–1153. 10.1016/j.patrec.2011.02.022

[B8] Cecotti H. Graser A. (2010). Convolutional neural networks for P300 detection with application to brain-computer interfaces. *IEEE Trans. Pattern Anal. Mach. Intell.* 33 433–445. 10.1109/TPAMI.2010.125 20567055

[B9] Chai X. Zhang Z. Guan K. Lu Y. Liu G. Zhang T. (2020). A hybrid bci-controlled smart home system combining ssvep and emg for individuals with paralysis. *Biomed. Signal Process. Control* 56:101687. 10.1016/j.bspc.2019.101687

[B10] Chen X. Wang Y. Gao S. Jung T. P. Gao X. (2015). Filter bank canonical correlation analysis for implementing a high-speed SSVEP-based brain–computer interface. *J. Neural Eng.* 12:046008. 10.1088/1741-2560/12/4/046008 26035476

[B11] Dehzangi O. Farooq M. (2018). Portable brain-computer interface for the intensive care unit patient communication using subject-dependent SSVEP identification. *BioMed Res. Int.* 2018:9796238. 10.1155/2018/9796238 29662908 PMC5832111

[B12] Dwarampudi M. Reddy N. V. (2019). Effects of padding on LSTMs and CNNs. *arXiv* [Preprint] arXiv:1903.07288 10.48550/arXiv.1903.07288

[B13] Farmaki C. Krana M. Pediaditis M. Spanakis E. Sakkalis V. (2019). “Single-channel SSVEP-based BCI for robotic car navigation in real world conditions,” in *Proceedings of the 2019 IEEE 19th International Conference on Bioinformatics and Bioengineering (BIBE)*, (Piscataway, NJ: IEEE), 638–643. 10.1109/BIBE.2019.00120

[B14] Jang E. Gu S. Poole B. (2016). Categorical reparameterization with gumbel-softmax. *arXiv* [Preprint] arXiv:1611.01144 10.48550/arXiv.1611.01144

[B15] Katzenbach J. R. Smith D. K. (2015). *The Wisdom Of Teams: Creating The High-Performance organization.* Boston, MA: Harvard Business Review Press.

[B16] Lawhern V. J. Solon A. J. Waytowich N. R. Gordon S. M. Hung C. P. Lance B. J. (2018). EEGNet: a compact convolutional neural network for EEG-based brain–computer interfaces. *J. Neural Eng.* 15:056013. 10.1088/1741-2552/aace8c 29932424

[B17] LeCun Y. Bengio Y. Hinton G. (2015). Deep learning. *Nature* 521 436–444. 10.1038/nature14539 26017442

[B18] LeCun Y. Bottou L. Bengio Y. Haffner P. (1998). Gradient-based learning applied to document recognition. *Proc. IEEE* 86 2278–2324. 10.1109/5.726791

[B19] Li Q. Lu Z. Gao N. Yang J. (2019). Optimizing the performance of the visual P300-speller through active mental tasks based on color distinction and modulation of task difficulty. *Front. Hum. Neurosci.* 13:130. 10.3389/fnhum.2019.00130 31057381 PMC6478661

[B20] Mary Judith A. Baghavathi Priya S. (2021). Multiset task related component analysis (M-TRCA) for SSVEP frequency recognition in BCI. *J. Ambient Intell. Hum. Comput.* 12 5117–5126. 10.1007/s12652-020-01962-8

[B21] Munzert J. Lorey B. Zentgraf K. (2009). Cognitive motor processes: the role of motor imagery in the study of motor representations. *Brain Res. Rev.* 60 306–326. 10.1016/j.brainresrev.2008.12.024 19167426

[B22] Nayak T. Ko L. W. Jung T. P. Huang Y. (2019). “Target classification in a novel SSVEP-RSVP based BCI gaming system,” in *Proceedings of the 2019 IEEE International Conference on Systems, Man and Cybernetics (SMC) (pp. 4194-4198)*, (Piscataway, NJ: IEEE), 10.1109/SMC.2019.8914174

[B23] Pan S. J. Yang Q. (2009). A survey on transfer learning. *IEEE Trans. Knowl. Data Eng.* 22 1345–1359. 10.1109/TKDE.2009.191

[B24] Peng Y. Wong C. M. Wang Z. Wan F. Vai M. I. Mak P. U. (2019). Fatigue evaluation using multi-scale entropy of EEG in SSVEP-based BCI. *IEEE Access* 7 108200–108210. 10.1109/ACCESS.2019.2932503

[B25] Ravi A. Beni N. H. Manuel J. Jiang N. (2020). Comparing user-dependent and user-independent training of CNN for SSVEP BCI. *J. Neural Eng.* 17:026028. 10.1088/1741-2552/ab6a67 31923910

[B26] Shao L. Zhang L. Belkacem A. N. Zhang Y. Chen X. Li J. (2020). EEG-controlled wall-crawling cleaning robot using SSVEP-based brain-computer interface. *J. Healthcare Eng.* 2020:6968713. 10.1155/2020/6968713 32399166 PMC7201509

[B27] Song X. Zeng Y. Tong L. Shu J. Yang Q. Kou J. (2022). A collaborative brain-computer interface framework for enhancing group detection performance of dynamic visual targets. *Comput. Intell. Neurosci.* 2022:4752450. 10.1155/2022/4752450 35087580 PMC8789438

[B28] Vaid S. Singh P. Kaur C. (2015). “EEG signal analysis for BCI interface: A review,” in *Proceedings 2015 fifth International Conference On Advanced Computing & Communication Technologies*, (Piscataway, NJ: IEEE), 143–147. 10.1109/ACCT.2015.72

[B29] Valeriani D. Poli R. Cinel C. (2016). Enhancement of group perception via a collaborative brain–computer interface. *IEEE Trans. Biomed. Eng.* 64 1238–1248. 10.1109/TBME.2016.2598875 28541187

[B30] Wang Y. Jung T. P. (2011). A collaborative brain-computer interface for improving human performance. *PLoS One* 6:e20422. 10.1371/journal.pone.0020422 21655253 PMC3105048

[B31] Wolpaw J. R. Birbaumer N. Heetderks W. J. McFarland D. J. Peckham P. H. Schalk G. (2000). Brain-computer interface technology: a review of the first international meeting. *IEEE Trans. Rehabil. Eng.* 8 164–173. 10896178 10.1109/tre.2000.847807

[B32] Zhang X. Xu G. Mou X. Ravi A. Li M. Wang Y. (2019). A convolutional neural network for the detection of asynchronous steady state motion visual evoked potential. *IEEE Trans. Neural Syst. Rehabil. Eng.* 27 1303–1311. 10.1109/TNSRE.2019.2914904 31071044

[B33] Zhang Z. Sabuncu M. R. (2018). Generalized cross entropy loss for training deep neural networks with noisy labels. In Proceedings of the 32nd International Conference on Neural Information Processing Systems. pp. 8792–8802. 10.48550/arXiv.1805.07836 PMC11747755 39839708

[B34] Zhao X. Chu Y. Han J. Zhang Z. (2016). SSVEP-based brain–computer interface controlled functional electrical stimulation system for upper extremity rehabilitation. *IEEE Trans. Syst. Man Cybern.* 46 947–956. 10.1109/TSMC.2016.2523762

