# Corrigendum: Multi-person feature fusion transfer learning-based convolutional neural network for SSVEP-based collaborative BCI

**DOI:** 10.3389/fnins.2022.1024150

**Published:** 2022-10-26

**Authors:** Penghai Li, Jianxian Su, Abdelkader Nasreddine Belkacem, Longlong Cheng, Chao Chen

**Affiliations:** ^1^School of Integrated Circuit Science and Engineering, Tianjin University of Technology, Tianjin, China; ^2^Department of Computer and Network Engineering, College of Information Technology, UAE University, Al Ain, United Arab Emirates; ^3^China Electronics Cloud Brain Technology Co., Ltd., Tianjin, China; ^4^Key Laboratory of Complex System Control Theory and Application, Tianjin University of Technology, Tianjin, China

**Keywords:** steady-state visually evoked potential, collaborative BCI, feature fusion, convolutional neural network, transfer learning

In the published article, there was an error in the article title as published. Instead of “Steady-state visually evoked potential collaborative BCI system deep learning classification algorithm based on multi-person feature fusion transfer learning-based convolutional neural network,” it should be “Multi-person feature fusion transfer learning-based convolutional neural network for SSVEP-based collaborative BCI.”

The authors apologize for this error and state that this does not change the scientific conclusions of the article in any way. The original article has been updated.

## Publisher's note

All claims expressed in this article are solely those of the authors and do not necessarily represent those of their affiliated organizations, or those of the publisher, the editors and the reviewers. Any product that may be evaluated in this article, or claim that may be made by its manufacturer, is not guaranteed or endorsed by the publisher.

